# Retraction: Targeting posttranslational modifications of RIOK1 inhibits the progression of colorectal and gastric cancers

**DOI:** 10.7554/eLife.108007

**Published:** 2025-06-18

**Authors:** Xuehui Hong, He Huang, Xingfeng Qiu, Zhijie Ding, Xing Feng, Yuekun Zhu, Huiqin Zhuo, Jingjing Hou, Jiabao Zhao, Wangyu Cai, Ruihua Sha, Xinya Hong, Yongxiang Li, Hongjiang Song, Zhiyong Zhang

**Keywords:** Human

Hong X, Huang H, Qiu X, Ding Z, Feng X, Zhu Y, Zhuo H, Hou J, Zhao J, Cai W, Sha R, Hong X, Li Y, Song H, Zhang Z. 2018. Targeting posttranslational modifications of RIOK1 inhibits the progression of colorectal and gastric cancers. *eLife* **7**:e29511. doi: 10.7554/eLife.29511.

Published 31 January 2018

Dr Zhiyong Zhang, on behalf of all the authors, is retracting this eLife publication in which they proposed that SETD7-mediated K411 methylation and CK2-mediated T410 phosphorylation dynamically regulate RIOK1 protein stability, and that these modifications control the aberrant progression of gastric cancers.

Duplication of images had been reported on PubPeer (https://pubpeer.com/publications/B49C40E8FC66A9E2118DB0D8F296EA), affecting Figures 2M, 5J, 6G, 7H, and 9H. Specifically, the SETD7 and Ub bands from Figure 5J duplicate the HA-ubiquitin and Myc bands of Figure 5K from He et al. 2017 (doi: 10.18632/oncotarget.15529). Secondly, the V5 band from Figure 6G duplicates the GST / RioK1 band from Figure 7H, which also duplicates the ATF4 band of Figure 4B from Zeng et al. 2016 (doi: 10.18632/oncotarget.6940). Lastly, the HE Staining panel from Figure 2M RKO / RioK1 overlaps with Figure 9H T410E+K411 R.

Upon review by the authors, the figure duplications were caused by errors in figure management and unfamiliarity with the software during figure preparation. Dr Zhang takes full responsibility for the errors and has provided the editorial office with detailed explanations and original records via multiple emails. Email correspondence with the corresponding authors of the two Oncotarget papers confirms that they mistakenly used the images for the eLife paper in their publications.

Dr Zhang independently repeated the in vitro and in vivo experiments related to the questioned figures three times, with all results consistent with the original conclusions. These additional data have also been submitted to the editorial office. However, given the concerns raised by the figure duplications and their impact on readers and co-authors, Dr Zhang, in consultation with the editorial office, has decided to retract the paper. All of the authors agree that retraction is the appropriate course of action.

